# Decoding the Factor H‐Related Proteins: Gatekeepers of Complement Dysregulation in AMD

**DOI:** 10.1002/eji.70228

**Published:** 2026-06-17

**Authors:** Jiaqi Tang, Nathalie Zgoda, Simon J. Clark

**Affiliations:** ^1^ Institute for Ophthalmic Research Department for Ophthalmology Eberhard Karls University of Tübingen Tübingen Germany; ^2^ University Eye Clinic Eberhard Karls University of Tübingen Tübingen Germany; ^3^ Lydia Becker Institute of Immunology and Inflammation Faculty of Biology, Medicine and Health University of Manchester Manchester UK

## Abstract

Age‐related macular degeneration (AMD) is the third most common form of blindness in the Western world, with a predicted 288 million individuals affected worldwide by the year 2040. Both genetic and biochemical evidence point toward complement overactivation, through a diminished regulatory capacity, at the back of the eye, causing inflammation and tissue damage that helps drive this devastating disease. While much historic effort has gone into understanding the loss of regulatory control by complement factor H and factor H‐like protein 1, recent studies have uncovered an emerging role of the factor H‐related proteins and their capacity for driving forward complement amplification. FHR gene deletions have been shown to be protective against AMD, and increased circulating levels of FHR proteins have been found to associate with their deposition in the back of the eye at the site of disease pathogenesis. Here, we will explore the current understanding of FHR and their association with AMD risk, possible mechanisms by which they promote inflammation and extracellular matrix remodeling, and the potential effectiveness of their targeting as a novel therapeutic strategy for reducing the risk of AMD.

## Graphical abstract

This review will explore the role of the factor H‐related (FHR) proteins in age‐related macular degeneration (AMD), their accumulation in the outer blood/retinal barrier, and how they may contribute to barrier dysfunction and inflammation.

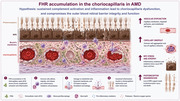

## 1 Introduction

The complement system is a central component of innate immunity that plays a critical role in host defense and immune surveillance [1]. It consists of a network of more than 30 individual proteins that can be activated through three main pathways: the classical, lectin, and alternative pathways [2]. The capacity for a complement response to cause immune cell recruitment, inflammation, and tissue remodeling is so potent that it requires strict regulation [3]. While the classical and lectin pathways represent targeted activation (through C1q or MBL, respectively), the alternative pathway (AP) acts as an amplification loop and can be spontaneously activated on biological surfaces through continuous low‐level hydrolysis of C3_(H2O)_. Activation of the alternative pathway leads to the formation of the C3 convertase (C3bBb), which further amplifies C3 cleavage and promotes downstream inflammatory responses, including C5 activation, membrane attack complex (MAC) formation, release of the anaphylatoxins C3a and C5a, and the recruitment of immune cells. Because uncontrolled complement activation may damage host tissues, several regulators tightly control the alternative pathway. One of the most important of these is complement factor I (FI), a protease that cleaves C3b into inactive C3b (iC3b), which cannot contribute to the ongoing amplification of complement activation [1]. FI further cleaves iC3b (which itself remains a potent opsonin) into smaller fragments C3dg and ultimately C3d. However, FI cannot do this alone and must have a cofactor present to ensure correct conformational engagement with C3b/iC3b/C3dg for proteolytic function. Cell membrane‐bound cofactors include membrane cofactor protein (MCP or CD46) and complement receptor 1 (CR1 or CD35). Conversely, acellular structures bereft of any such cofactors, for example, the extracellular matrix (ECM) and glycocalyx, remain vulnerable to complement activation and rely solely on soluble FI cofactors such as complement factor H (FH) and factor H‐like protein 1 (FHL‐1). The immobilization of these soluble cofactors to the ECM through their interactions with glycosaminoglycans (such as heparan sulphate [HS]) and sialic acid allows them to act as surrogate membrane‐bound regulators and are vital to their function in protecting acellular structures from unwarranted complement attack.

**FIGURE 1:**
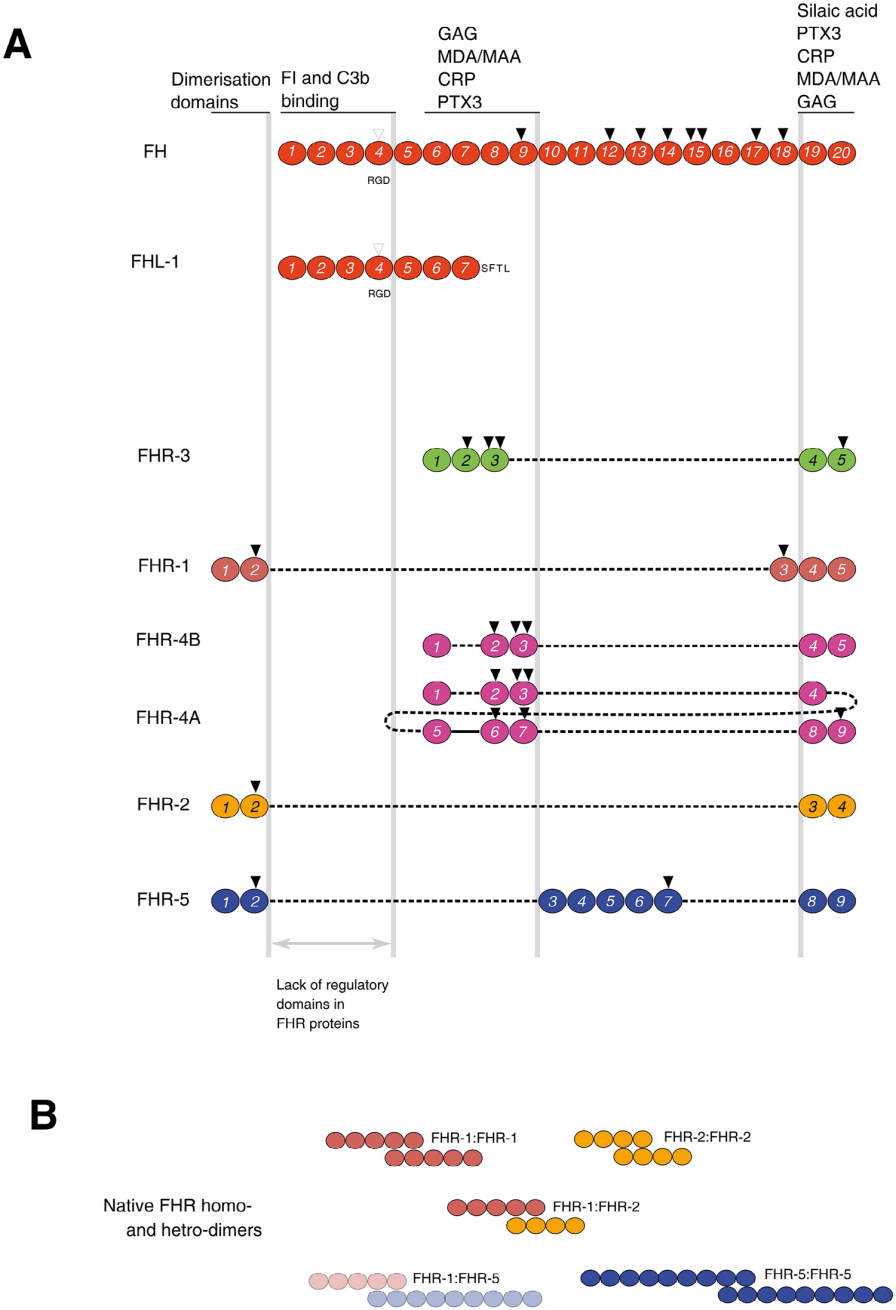
The protein structures of the factor H‐related protein family. (A) The protein structures arising from the *CFH/CFHR* genes with their CCP domains aligned and compared with the FH protein in terms of functional similarities. The RGD integrin binding motif in FH/FHL‐1 in CCP4 and the unique C‐terminal tail sequence of FHL‐1 (i.e., SFTL) are highlighted. q symbolizes the presence of occupied N‐linked glycosylation sites, and s the unoccupied glycosylation site in FHL‐1. (B) A schematic representation of the native FHR protein homo‐ and heterodimers believed to be present in the circulation. However, a recent report suggests that FHR‐5 only forms homodimers in the circulation [5]. Each protein dimerises through its N‐terminal dimerization regions in CCPs 1 and 2 in the orientation shown.

Alongside FH and FHL‐1 are a series of additional soluble proteins called the factor H‐related proteins (FHRs). Transcribed by five distinct genes immediately downstream of the *CFH* gene on chromosome 1, six proteins can be found circulating in human blood: FHR‐1, FHR‐2, FHR‐3, FHR‐4A, FHR‐4B, and FHR‐5 (see Figure 1), although FHR‐4A is the predominant splice variant of the *CFHR4* gene found in circulation [4]. The six FHR proteins expressed by the five *CFHR* genes can be divided into two groups: FHRs (including FHR‐1, FHR‐2, and FHR‐5), which exist as homo‐ and heterodimers; and FHRs (FHR‐3, FHR‐4A, and FHR‐4B), which exist as monomers [5, 6]. The role of FHR proteins in complement regulation appears to change depending on the context in which they are found, but in many circumstances, they are believed to compete with FH/FHL‐1 for either C3b binding or HS binding. Importantly, none of the FHR proteins can bind FI, so this competitive binding ultimately leads to dysregulation of complement on a surface, and therefore, the FHR proteins are generally considered complement activators, or at least modifiers of regulation.

## 2 Structure and Evolution of the FHR Family of Proteins

Most of the genes encoding regulators of the complement pathway reside in a 12 Mb region of DNA on chromosome 1 (1q31‐1q32) referred to as the region of complement activation (RCA) cluster [7]. It comprises two gene islands separated by a 10.3 Mb region of DNA that, while itself containing transcribed genes, does not contain genes related to the complement system. The telomeric 707 kb‐long DNA section contains numerous fluid phase and membrane‐bound complement regulators (such as *C4BPA*, *C4BPB*, *DAF*, *CR1*, *CR2*, etc.), but it is the 358 kb‐long centromeric DNA section that contains the *CFH* and five *CFHR* genes. The RCA cluster itself was originally mapped in 1999 [8] and later refined in 2001 [9] to become the version mostly used today. Admittedly, the complexity of this genetic region and the advent of new sequencing technologies mean that our understanding of these regions’ structural make‐up continues to evolve over time [10], and the possibility of discovering novel copy number and sequence variations remains.

Each of the *CFH* and five *CFHR* genes transcribes its own proteins, with the exception of *CFH* and *CFHR4*, which both transcribe additional alternative splicing variants. The *CFH* gene was found to produce not only an mRNA species of 4.3 kb associated with the full‐length 155 kDa FH protein, but also an mRNA species of 1.8 kb [11, 12]. This smaller truncated form of the *CFH* transcript is spliced to a unique exon, which encodes a unique four C‐terminal amino acid tail, a stop codon, and a unique untranslated 3′ end [13]. When translated, this transcript was found to correspond to a 49.1 kDa portion of the N‐terminus region of FH [14, 15] that contained the required C3b and FI binding regions that confer complement regulatory function. The truncated transcript was found to terminate in a unique sequence that corresponded to a unique four‐amino acid C‐terminal tail in the resulting truncated protein (Ser‐Phe‐Thr‐Leu), which was later termed factor H‐like protein 1 (FHL‐1) [16]. The *CFHR4* gene also has two mRNA transcripts, which lead to the production of two proteins, FHR‐4A and FHR‐4B [17, 18]. Very little is known about the exact mechanisms deciding the splicing event of the *CFH* gene to give either FH or FHL‐1, and despite the larger FH protein being predominantly expressed almost ubiquitously throughout the body, there are rare examples of where cells make more FHL‐1 than FH, such as in glioblastoma cells [19] and the ovarian tumor cell lines SK‐OV‐3 and Caov‐3 [20].

## 3 Complement Activation and Its Association With AMD

**FIGURE 2:**
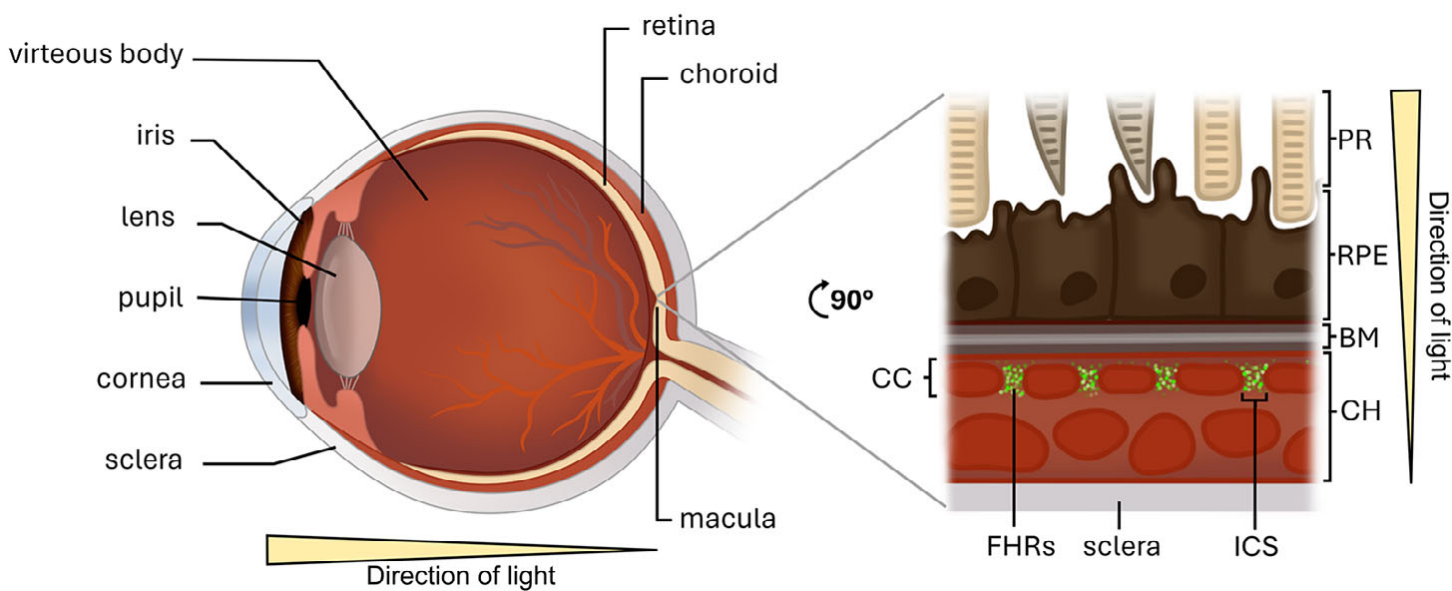
Schematic of the human eye and location of FHR accumulation. (A) Age‐related macular degeneration manifests as the progressive destruction of the macula, the central region of the retina at the back of the eye that is responsible for central visual acuity. (B) The outer blood/retinal barrier comprises the retinal pigment epithelium (RPE) and specialist ECM Bruch's membrane (BM). This barrier separates the photoreceptors (PR) from the choroid (CH), the main source of systemic blood flow in the eye. Underlying the BM is the fenestrated blood vasculature called the choriocapillaris (CC) from which the RPE and PR receive essential nutrients. The ECM surrounding the CC forms distinct pillars separating the vasculature, referred to as the intercapillary septa (ICS). It is within the ICS that the FHR proteins have been found to accumulate and co‐localize with increased C3b deposition in donor eyes from AMD patients.

The dysregulation of complement has been associated with several systemic and organ‐specific diseases [21]. But recently, a major area of interest lies in a common form of blinding disease, called age‐related macular degeneration (AMD) [22]. AMD manifests at the back of the human eye, with the gradual destruction of the macular region of the retina responsible for central vision (see Figure 2) [23], and is predicted to affect over 288 million individuals by the year 2040 [24]. Since the late 1990's studies had noted that, in postmortem eyes from donors who suffered AMD, there was a significant deposition of C3b and other complement proteins in and around the sites of disease activation, namely the choriocapillaris (the fenestrated blood vasculature underlying the outer blood/retinal barrier) and Bruch's membrane (a specialist ECM that forms one half of the outer blood/retinal barrier along with the retinal pigment epithelium [RPE]) [23].

As the third leading cause of blindness in the world, AMD has been the subject of several Genome Wide Association studies (GWAS) that have ultimately led to the identification of 52 independently associated common and rare genetic risk variants covering 34 loci [25]. Of these, there are two vastly predominant risk loci: one on chromosome 10 around the *HTRA1/ARMS2* genes, and the other on chromosome 1 focusing on the gene encoding FH and FHL‐1 (*CFH*). Genetic variants in these two risk loci have the highest single attributable risk scores for AMD [25], where one single nucleotide polymorphism (SNP) in the *CFH* gene (rs1061170) is believed to account for ∼50% of attributable risk for AMD [26]. This SNP tags a common polymorphism in both the FH and FHL‐1 proteins, where a histidine residue replaces a tyrosine residue at position 402 (position 384 in the mature secreted proteins [27]), referred to as the Y402H polymorphism. It should be noted that this polymorphism exists as part of a risk haplotype, which includes multiple other SNPs in and around the *CFH* gene, all collectively contributing to the risk [28]. Nevertheless, it is intriguing to note that this Y402H polymorphism alone is sufficient to alter the recognition of HS on ECM, such as Bruch's membrane [29], a major component of the outer blood/retinal barrier in the eye (see Figure 1), and the site of early changes in AMD [23].

## 4 The Association of FHR Proteins With AMD

To attribute all genetic risk for AMD at chromosome 1 solely to functional changes to the FH/FHL‐1 proteins would be disingenuous. Dual deletions of the *CFHR1/CFHR3* or *CFHR1/CFHR4* genes are strongly associated with protection against the risk of developing AMD [30], where the protective effect is believed to be independent of, and stronger than, the risk conferred by the *CFH* Y402H polymorphism [31]. Some studies suggest that the protective effect of the *CFHR1/CFHR3* deletion is independent of *CFH* Y402H [31], whereas others report that the deletion does not segregate perfectly with any single SNP but instead occurs predominantly on protective *CFH* haplotypes and may only partially account for the reduced AMD risk observed at this locus [32, 33].

Consequently, it was discovered that increased circulating levels of FHR proteins in patients’ blood are also strongly associated with AMD [34, 35, 36, 37]. These elevated levels have been found to be genetically driven [35, 38], and using the GWAS study datasets, Mendelian‐randomization analysis strongly suggests that genetically elevated circulating levels of FHR‐1, FHR‐2, FHR‐4, and FHR‐5 are causative for disease [35]. Additionally, the FHR‐2, FHR‐4, and FHR‐5 proteins have been shown to accumulate in the intercapillary septa of the choriocapillaris in human eye tissues (Figure 2) [36, 37, 38]: the physiologically relevant context for the associated overactivation of complement in the early stages of AMD [39, 40, 41]. Recent studies have provided even more genetic and biochemical data, adding weight to the role of FHR‐1, and particularly FHR‐5, in driving inflammation at the back of the eye in AMD [42, 43, 44].

There are studies showing genetic risk variants incorporating the *CFH* and *CFHR* genes also lead to detectable complement activation products within the systemic circulation [45], although discussions remain around whether this represents systemic complement activation in AMD, or detection of local, organ‐specific, complement activation systemically. Similarly, some debate remains about the origin of FHR proteins accumulating in the choriocapillaris, that is, whether from a local or systemic source. Currently, the predominant source of any FHR protein is the liver [46]. Numerous studies have not seen any evidence of *CFHR* gene transcription within cells of the retina [47, 48, 49, 50, 51, 52] (see also the Human Eye Transcriptome Atlas at https://www.eye‐transcriptome.com/index.php), nor the presence of any FHR protein within the retinal space of human donor eyes beyond the outer blood‐retinal barrier [37]. However, FHR‐3 has been detected in the microglia in a single aged donor eye [34], and the exogenous application of recombinant FHR‐3 on ARPE‐19 cells in vitro led to the protein's internalization and enhanced secretion of pro‐inflammatory cytokines IL‐1ß, IL‐18, and complement proteins [53]. Clearly, if any FHR proteins do gain access to the retinal space, this would be deleterious to the health and homeostasis of the retina and a driver of a range of inflammatory diseases. Given that these predominantly liver‐sourced proteins accumulate in the ECM surrounding the choriocapillaris in the back of the eye, there must be a specific recognition of something within the ECM to make them “stick”. While this exact mechanism remains under investigation, it seems reasonable that they recognize clusters of GAG chain sequences which promote their immobilization [54]: a mechanism of sugar sequence‐mediated protein sequestration and specific localization referred to as the GAG chain ZIP code [55, 56].

A recent study utilizing patient‐derived induced pluripotent stem cell RPEs (iPSC‐RPE) demonstrated that exogenous complement activation led to RPE‐cell stimulation through the C3a/C3aR and C5a/C5aR axis, altering the NF‐κB activation and downregulated autophagy, and resulting in a series of AMD‐associated morphologies [57]. The concluding hypothesis was that extracellular complement activation affected the iPSC‐RPE cells in a genetic‐risk‐specific manner, where the iPSC‐RPE cells carrying the most genetic risk for AMD were more susceptible to C3aR and C5aR stimulation. This gives significant weight to the notion that inappropriate complement overactivation, driven perhaps by a shift in the FH/FHL‐1 and FHR functional balance within the local microenvironment, can drive disease pathogenesis.

## 5 FHR‐Mediated Complement Dysregulation in AMD

To best understand the biochemical consequences of FHR protein accumulation at the outer blood/retinal barrier, it is important to remember that due to its relatively small size and lack of glycosylation (the single predicted N‐linked glycosylation site in CCP4 remains unoccupied [58]) FHL‐1 has been found to be the predominant fluid‐phase regulator in Bruch's membrane, whereas the full length FH protein is unable to penetrate the same space [59]. This subtle detail is important because it means the levels of FHR proteins required to disrupt FHL‐1 recruitment to ECM and/or binding to C3b will probably be less than required to dysregulate the full‐length FH protein with its additional GAG binding site (CCPs 19–20), multiple C3b binding domains (CCPs 1–4 and CCPs 19–20), and its ability to recognize sialic acid (CCP 20) [60].

Heparin is a highly sulphated soluble GAG found predominantly released from activated mast cells, and unlike the other GAG classes (with the exception of the nonsulphated hyaluronan) is not found bound to a protein core to form a proteoglycan [61]. It is, however, commonly used in vitro as a model for the highly sulphated regions of HS, which is a major component of basement membranes and the glycocalyx that lines the lumen of blood vessels [62] and a major binding ligand for FH/FHL‐1 [63]. Of the six FHR proteins expressed by the five *CFHR* genes, FHRs 1, 3, and 5 have been shown to bind to heparin [6, 64, 65, 66]. FH, FHR‐1, and FHR‐5 bind HS in a sulphation‐dependent manner, with FHR‐1 preferentially recognizing N‐sulphated HS and outcompeting HS‐bound FH, suggesting that local FHR‐to‐FH/FHL‐1 ratios shape tissue complement regulation [67]. It is interesting to note that there is a significant level of N‐sulphated HS GAGs present in Bruch's membrane in the human eye [68]. Indeed, the specificity of GAG sulphation patterns to which FHRs bind in the kidney glycocalyx has been noted in C3 glomerulopathy, such that the authors have proposed 2‐O‐desulfated heparin derivatives as potential therapeutics for disrupting FHR‐1 and FHR‐5 deposition without affecting FH [67].

Aside from FHR proteins interfering with surface immobilization through HS interactions and directly altering FH/FHL‐1 levels in tissues, other functions have been identified to alter complement activation. Early studies suggested that FHR‐3 and FHR‐4 themselves could act as FI cofactors and mediate C3b breakdown [31, 64], although these were performed under nonphysiologically relevant conditions. Conversely, subsequent studies failed to replicate the FHR‐4 data and saw no cofactor activity [69]. These latter results are supported by the domain homology shared between the FHR proteins and FH/FHL‐1, whereby the FI cofactor regions are absent in the FHR proteins, implying they are unable to facilitate FI interactions (see Figure 1). FHR‐2 has been found to bind C3b and inhibit the activity of C3‐convertase, effectively acting as a complement inhibitor [70], and it has been suggested that FHR‐1 is able to inhibit the formation of the MAC complex [71]. However, most of these results have been disputed and/or difficult to replicate under physiological conditions [72, 73]. More recent studies all appear to agree that one way or another, the binding of FHR proteins to C3b drives forward complement activation in direct contrast to FH and FHL‐1. One proposed mechanism is through their competition for FH/FHL‐1 binding to C3b [69]: FHR‐4 is known to outcompete both FHL‐1 and, to a lesser extent, FH to immobilized C3b [36]. Indeed, this exact property of FHR‐4 has been weaponized by the protein's inclusion in immunoconjugates and their delivery to HER‐2 positive tumor cells to overcome the tumors’ complement resistance [74]. The FHR proteins have also been shown to bind PTX‐3 and CRP, with FHR‐5 outcompeting FH binding to PTX3 [75], and FHR‐1 and FHR‐4 were shown to activate the classical pathway through binding to CRP [69, 72, 76]. It is likely that the complete array of FHR protein ligands, and the exact subsequent biochemical consequences of their binding, are yet to be discovered, but the considerable current evidence supports the notion that the FHR proteins are activators of complement and work in balance with the negative regulators FH and FHL‐1. A recent study has investigated what the soluble FHR proteins may be interacting with in the blood as they travel from the liver to various sites around the body [66]. This study identified a series of novel blood‐borne interactors, including C4, cathepsin G, and MBL2, all of which have in some way been linked with ECM remodeling and inflammation in AMD.

## 6 Therapeutic Modulation

Given the role of FH, FHL‐1, and FHR proteins in disease, it seems unsurprising that attempts to modify their functions in vivo are being considered as therapeutic avenues for various specific diseases, which are better described elsewhere [77, 78]. In AMD, FH and FHL‐1 supplementation, by either intravitreal injections or gene therapy, are attempting to regain control of a runaway complement amplification loop. However, it remains to be seen if these strategies infiltrate the right tissues and mitigate the effects of increased C3b deposition in the presence of increased FHR protein competition. Indeed, the ability of FHR‐4 to out‐compete FH/FHL‐1 binding for C3b is being used to direct immunoconjugates to HER‐2 positive tumor cells [74]. This is, in fact, a striking effect given that these proteins do not appear to bind to the same primary sites on C3b but could nevertheless perhaps sterically hinder each other's binding.

The strong genetic and biochemical evidence linking the FHR proteins to disease makes them, in their own right, potential targets for therapeutic interventions in disease. However, their high level of sequence homology has been a barrier to the development of reliable detection reagents for so long [73], and also poses a problem for their specific therapeutic targeting. Given that the main source of *CFHR* gene transcription is in the human liver [36], anyone wishing to target the FHR proteins either must do this in the circulation, which itself poses dosing and pharmacokinetic hurdles, or direct targeting of the liver itself. Precedence for the latter route of administration has been set with programs targeting the gene transcription of other complement proteins (Factor B) in the liver in an attempt to lower their circulating concentrations [phase II clinical trial NCT03815825]. However, preclinical in vivo modeling remains a significant challenge as the proposed mouse FHR protein homologues may share some functional similarities [79] but are unlikely to replicate all the functional and tissue‐specific features of human FHR proteins. Nonhuman primate models of disease, which likely include better FHR protein homologues and thus the only real legitimate way of showing in vivo efficacy, remain out of reach for the majority of academic researchers. To date, the authors of this review are unaware of any clinical trials pursuing the targeting of FHR proteins in disease, but are aware through personal communications that this will not remain the case for long.

## 7 Conclusions and Future Perspectives

There is no doubt that, while we know more regarding the extended factor H family of proteins, there remains more to be discovered. The RCA gene cluster maps are being constantly refined, and genetic variants within the RCA cluster are associated with more diseases, both rare and common. It remains to be seen if targeting the FH‐family of proteins can bring about any real efficacious benefit for patients in the clinic, but there appear to be plenty of opportunities to try. After decades of focusing on the functional role of FH in isolation, perhaps it is time to think beyond factor H and include the other members of the FH family of proteins when considering mechanisms driving immune surveillance and regulation.

## Author Contributions

J.T. and S.J.C. conceived and drafted the manuscript. N.Z. created the figures. J.T., N.Z., and S.J.C. revised manuscript content and format.

## Conflicts of Interest

S.J.C. is an inventor named in patent applications that describe the use of complement inhibitors for therapeutic purposes and the use of circulating complement protein measurements for patient stratification, and is a co‐founder and shareholder of Complement Therapeutics, a company that focuses on the development of complement‐targeted therapeutics, including for AMD. J.T. and N.Z. declare no conflicts of interest.

## Data Availability

Data sharing not applicable to this article as no datasets were generated or analyzed during the current study.

## References

[1] M. J. Walport , “Complement. First of Two Parts,” New England Journal of Medicine 344 (2001): 1058–1066, 10.1056/NEJM200104053441406. 11287977

[2] D. Ricklin , E. S. Reis , and J. D. Lambris , “Complement in Disease: A Defence System Turning Offensive,” Nature Reviews Nephrology 12 (2016): 383–401, 10.1038/nrneph.2016.70. 27211870 PMC4974115

[3] N. S. Merle , S. E. Church , V. Fremeaux‐Bacchi , and L. T. Roumenina , “Complement System Part I—Molecular Mechanisms of Activation and Regulation,” Frontiers in immunology 6 (2015): 262, 10.3389/fimmu.2015.00262. 26082779 PMC4451739

[4] R. B. Pouw , M. C. Brouwer , A. E. van Beek , M. Józsi , D. Wouters , and T. W. Kuijpers , “Complement Factor H‐Related Protein 4A Is the Dominant Circulating Splice Variant of CFHR4,” Frontiers in immunology 9 (2018): 729, 10.3389/fimmu.2018.00729. 29719534 PMC5913293

[5] A. E. van Beek , R. B. Pouw , M. C. Brouwer , et al., “Factor H‐Related (FHR)‐1 and FHR‐2 Form Homo‐ and Heterodimers, While FHR‐5 Circulates Only as Homodimer in human Plasma,” Frontiers in immunology 8 (2017): 1328, 10.3389/fimmu.2017.01328. 29093712 PMC5651247

[6] E. Goicoechea de Jorge , J. J. E. Caesar , T. H. Malik , et al., “Dimerization of Complement Factor H‐related Proteins Modulates Complement Activation in Vivo,” Proceedings of the National Academy of Sciences 110 (2013): 4685–4690, 10.1073/pnas.1219260110. PMC3606973 23487775

[7] S. R. De Cordoba , D. M. Lublin , P. Rubinstein , and J. P. Atkinson , “Human Genes for Three Complement Components That Regulate the Activation of C3 Are Tightly Linked,” Journal of Experimental Medicine 161 (1985): 1189–1195, 10.1084/jem.161.5.1189. 3157763 PMC2187593

[8] S. R. De Cordoba , M. A. Díaz‐Guillén , and D. Heine‐Suñer , “An Integrated Map of the Human Regulator of Complement Activation (RCA) Gene Cluster on 1q32,” Molecular Immunology 36 (1999): 803–808, 10.1016/S0161-5890(99)00100-5. 10698333

[9] D. Pérez‐Caballero , C. González‐Rubio , M. E. Gallardo , M. Vera , M. López‐Trascasa , and P. Sánchez‐Corral , “Clustering of Missense Mutations in the C‐Terminal Region of Factor H in Atypical Hemolytic Uremic Syndrome,” American Journal of Human Genetics 68 (2001): 478–484, 10.1086/318201. 11170895 PMC1235280

[10] J. García‐Fernández , S. Vilches‐Arroyo , L. Olavarrieta , J. Pérez‐Pérez , and S. Rodríguez de Córdoba , “Detection of Genetic Rearrangements in the Regulators of Complement Activation RCA Cluster by High‐Throughput Sequencing and MLPA,” Methods in Molecular Biology 2227 (2021): 159–178, 10.1007/978-1-0716-1016-9_16. 33847941

[11] A. J. Day , J. Ripoche , A. Lyons , B. McIntosh , T. J. R. Harris , and R. B. Sim , “Sequence Analysis of a cDNA Clone Encoding the C‐Terminal End of Human Complement Factor H,” Bioscience Reports 7 (1987): 201–207, 10.1007/BF01124790. 2889480

[12] W. Schwaeble , J. Zwirner , T. F. Schulz , R. P. Linke , M. P. Dierich , and E. H. Weiss , “Human Complement Factor H: Expression of an Additional Truncated Gene Product of 43 kDa in human Liver,” European Journal of Immunology 17 (1987): 1485–1489, 10.1002/eji.1830171015. 2445583

[13] R. B. Sim , K. Kölble , M. A. Mcaleer , O. Dominguez , and V. M. Dee , “Genetics and Deficiencies of the Soluble Regulatory Proteins of the Complement System,” International Reviews of Immunology 10 (1993): 65–86, 10.3109/08830189309051172. 8340678

[14] M. Fontaine , M. J. Demares , V. Koistinen , C. Davrinche , R. B. Sim , and J. Ripoche , “Truncated Forms of Human Complement Factor H,” Biochemical Journal 258 (1989): 927–930, 10.1042/bj2580927. 2525027 PMC1138455

[15] J. Ripoche , A. J. Day , T. J. Harris , and R. B. Sim , “The Complete Amino Acid Sequence of Human Complement Factor H,” Biochemical Journal 249 (1988): 593–602, 10.1042/bj2490593. 2963625 PMC1148743

[16] C. Estaller , W. Schwaeble , M. Dierich , and E. H. Weiss , “Human Complement Factor H: Two Factor H Proteins Are Derived From Alternatively Spliced Transcripts,” European Journal of Immunology 21 (1991): 799–802, 10.1002/eji.1830210337. 1826264

[17] C. Skerka , J. Hellwage , W. Weber , et al., “The Human Factor H‐Related Protein 4 (FHR‐4): A Novel Short Consensus Repeat‐Containing Protein is Associated With Human Triglyceride‐Rich Lipoproteins,” Journal of Biological Chemistry 272 (1997): 5627–5634, 10.1074/jbc.272.9.5627. 9038172

[18] M. Józsi , H. Richter , I. Löschmann , et al., “FHR‐4A: A New Factor H‐Related Protein Is Encoded by the Human FHR‐4 Gene,” European Journal of Human Genetics 13 (2005): 321–329, 10.1038/sj.ejhg.5201324. 15562282

[19] S. Junnikkala , T. S. Jokiranta , M. A. Friese , H. Jarva , P. F. Zipfel , and S. Meri , “Exceptional Resistance of Human H2 Glioblastoma Cells to Complement‐Mediated Killing by Expression and Utilization of Factor H and Factor H‐Like Protein 1,” The Journal of Immunology 164 (2000): 6075–6081, 10.4049/jimmunol.164.11.6075. 10820293

[20] S. Junnikkala , J. Hakulinen , H. Jarva , et al., “Secretion of Soluble Complement Inhibitors Factor H and Factor H‐Like Protein (FHL‐1) by Ovarian Tumour Cells,” British Journal of Cancer 87 (2002): 1119–1127, 10.1038/sj.bjc.6600614. 12402151 PMC2376183

[21] R. Parente , S. J. Clark , A. Inforzato , and A. J. Day , “Complement Factor H in Host Defense and Immune Evasion,” Cellular and Molecular Life Sciences 74 (2017): 1605–1624, 10.1007/s00018-016-2418-4. 27942748 PMC5378756

[22] A. Armento , M. Ueffing , and S. J. Clark , “The Complement System in Age‐Related Macular Degeneration,” Cellular and Molecular Life Sciences 1 (2021): 3, 10.1007/s00018-021-03796-9. PMC8195907 33751148

[23] M. Fleckenstein , S. Schmitz‐Valckenberg , and U. Chakravarthy , “Age‐Related Macular Degeneration: A Review,” Jama 331 (2024): 147–157, 10.1001/jama.2023.26074. 38193957 PMC12935482

[24] W. L. Wong , X. Su , X. Li , et al., “Global Prevalence of Age‐Related Macular Degeneration and Disease Burden Projection for 2020 and 2040: A Systematic Review and Meta‐Analysis,” The Lancet Global Health 2 (2014): e106–e116, 10.1016/S2214-109X(13)70145-1. 25104651

[25] L. G. Fritsche , W. Igl , J. N. C. Bailey , et al., “A Large Genome‐Wide Association Study of Age‐Related Macular Degeneration Highlights Contributions of Rare and Common Variants,” Nature Genetics 48 (2016): 134–143, 10.1038/ng.3448. 26691988 PMC4745342

[26] A. O. Edwards , R. Ritter , K. J. Abel , A. Manning , C. Panhuysen , and L. A. Farrer , “Complement Factor H Polymorphism and Age‐Related Macular Degeneration,” Science 308 (2005): 421–424, 10.1126/science.1110189. 15761121

[27] A. J. Day , A. C. Willis , J. Ripoche , and R. B. Sim , “Sequence Polymorphism of Human Complement Factor H,” Immunogenetics 27 (1988): 211–214, 10.1007/BF00346588. 2962936

[28] S. Raychaudhuri , O. Iartchouk , K. Chin , et al., “A Rare Penetrant Mutation in CFH Confers High Risk of Age‐Related Macular Degeneration,” Nature Genetics 43 (2011): 1232–1236, 10.1038/ng.976. 22019782 PMC3225644

[29] S. J. Clark , R. Perveen , S. Hakobyan , et al., “Impaired Binding of the Age‐Related Macular Degeneration‐Associated Complement Factor H 402H Allotype to Bruch's Membrane in Human Retina,” Journal of Biological Chemistry 285 (2010): 30192–30202, 10.1074/jbc.M110.103986. 20660596 PMC2943316

[30] A. E. Hughes , N. Orr , H. Esfandiary , M. Diaz‐Torres , T. Goodship , and U. Chakravarthy , “A Common CFH Haplotype, With Deletion of CFHR1 and CFHR3, Is Associated With Lower Risk of Age‐Related Macular Degeneration,” Nature Genetics 38 (2006): 1173–1177, 10.1038/ng1890. 16998489

[31] L. G. Fritsche , N. Lauer , A. Hartmann , et al., “An Imbalance of Human Complement Regulatory Proteins CFHR1, CFHR3 and Factor H Influences Risk for Age‐Related Macular Degeneration (AMD),” Human Molecular Genetics 19 (2010): 4694–4704, 10.1093/hmg/ddq399. 20843825

[32] K. L. Spencer , M. A. Hauser , L. M. Olson , et al., “Deletion of CFHR3 and CFHR1 Genes in Age‐Related Macular Degeneration,” Human Molecular Genetics 17 (2008): 971–977, 10.1093/hmg/ddm369. 18084039

[33] S. Raychaudhuri , S. Ripke , M. Li , et al., “Associations of CFHR1‐CFHR3 Deletion and a CFH SNP to Age‐Related Macular Degeneration Are Not Independent,” Nature Genetics 42 (2010): 553–555, 10.1038/ng0710-553. 20581873 PMC3138072

[34] N. Schafer , A. Grosche , J. Reinders , et al., “Complement Regulator FHR‐3 Is Elevated Either Locally or Systemically in a Selection of Autoimmune Diseases,” Frontiers in immunology 7 (2016): 542, 10.3389/fimmu.2016.00542. 27965669 PMC5124756

[35] V. Cipriani , A. Tierney , J. R. Griffiths , et al., “Beyond Factor H: The Influence of Genetic Variation With Age‐Related Macular Degeneration on Circulating Factor H‐Like 1 and Factor H‐Related Protein Levels,” American Journal of Human Genetics 108 (2021): p1385–1400, 10.1016/J.AJHG.2021.05.015. PMC8387294 34260948

[36] V. Cipriani , L. Lorés‐Motta , F. He , et al., “Increased Circulating Levels of Factor H‐Related Protein 4 Are Strongly Associated With Age‐Related Macular Degeneration,” Nature Communications 11 (2020): 778, 10.1038/s41467-020-14499-3. PMC7005798 32034129

[37] L. Lorés‐Motta , A. E. van Beek , E. Willems , et al., “Common Haplotypes at the CFH Locus and Low‐Frequency Variants in CFHR2 and CFHR5 Associate With Systemic FHR Concentrations and Age‐Related Macular Degeneration,” The American Journal of Human Genetics 108 (2021): 1367–1384, 10.1016/J.AJHG.2021.06.002. 34260947 PMC8387287

[38] M. A. Zouache , B. T. Richards , C. M. Pappas , et al., “Levels of Complement Factor H‐Related 4 Protein Do Not Influence Susceptibility to Age‐Related Macular Degeneration or Its Course of Progression,” Nature Communications 15 (2024): 1–17, 10.1038/s41467-023-44605-0. PMC10781981 38200010

[39] S. J. Clark and P. N. Bishop , “The Eye as a Complement Dysregulation Hotspot,” Seminars in Immunopathology 40 (2018): 65–74, 10.1007/s00281-017-0649-6. 28948331 PMC5794836

[40] D. L. Forest , L. V. Johnson , and D. O. Clegg , “Cellular Models and Therapies for Age‐Related Macular Degeneration,” DMM Disease Models and Mechanisms 8 (2015): 421–427, 10.1242/dmm.017236. 26035859 PMC4415892

[41] T. D. L. Keenan , M. Toso , C. Pappas , L. Nichols , P. N. Bishop , and G. S. Hageman , “Assessment of Proteins Associated With Complement Activation and Inflammation in Maculae of Human Donors Homozygous Risk at Chromosome 1 CFH‐to‐F13B,” Investigative Ophthalmology & Visual Science 56 (2015): 4870–4879, 10.1167/iovs.15-17009. 26218915

[42] M. P. Reeve , S. Loomis , E. Nissilä , et al., “Loss of CFHR5 Function Reduces the Risk for Age‐Related Macular Degeneration,” Nature Communications 16 (2025): 5766, 10.1038/S41467-025-61193-3;TECHMETA. PMC12217273 40593839

[43] A. Sekulic , S. M. Herr , K. Mulfaul , et al., “Factor‐H‐Related Protein 1 (FHR1), a Promotor of Para‐Inflammation in Age‐Related Macular Degeneration,” Journal of Neuroinflammation 22 (2025): 173, 10.1186/S12974-025-03499-Z/FIGURES/11. 40611130 PMC12226897

[44] M. Choudhary , E. N. Ismail , P. L. Yao , et al., “LXRs Regulate Features of Age‐Related Macular Degeneration and May be a Potential Therapeutic Target,” JCI Insight 5 (2020): e131928, 10.1172/jci.insight.131928. 31829999 PMC7030875

[45] L. Lorés‐Motta , C. C. Paun , J. Corominas , et al., “Genome‐Wide Association Study Reveals Variants in CFH and CFHR4 Associated With Systemic Complement Activation: Implications in Age‐Related Macular Degeneration,” Ophthalmology 125 (2018): 1064–1074, 10.1016/j.ophtha.2017.12.023. 29398083

[46] K. G. Ardlie , D. S. DeLuca , A. V. Segrè , et al., “The Genotype‐Tissue Expression (GTEx) Pilot Analysis: Multitissue Gene Regulation in Humans,” Science 348 (2015): 648–660, 10.1126/science.1262110. 25954001 PMC4547484

[47] W. Lagrèze , H. Agostini , and T. Reinhard , “The Human Eye Transcriptome Atlas: A Searchable Comparative Transcriptome Database for Healthy and Diseased Human Eye Tissue,” bioRxiv 10.1101/2021.11.04.467318. 35124170

[48] A. H. Wagner , V. N. Anand , W. H. Wang , et al., “Exon‐Level Expression Profiling of Ocular Tissues,” Experimental Eye Research 111 (2013): 105–111, 10.1016/j.exer.2013.03.004. 23500522 PMC3664108

[49] E. J. Kim , G. R. Grant , A. S. Bowman , N. Haider , H. V. Gudiseva , and V. R. M. Chavali , “Complete Transcriptome Profiling of Normal and Age‐Related Macular Degeneration Eye Tissues Reveals Dysregulation of Anti‐Sense Transcription,” Scientific Reports 8 (2018): 1–13, 10.1038/s41598-018-21104-7. 29445097 PMC5813239

[50] M. Li , C. Jia , K. L. Kazmierkiewicz , et al., “Comprehensive Analysis of Gene Expression in Human Retina and Supporting Tissues,” Human Molecular Genetics 23 (2014): 4001–4014, 10.1093/hmg/ddu114. 24634144 PMC7297232

[51] S. S. Whitmore , T. A. Braun , J. M. Skeie , et al., “Altered Gene Expression in Dry Age‐Related Macular Degeneration Suggests Early Loss of Choroidal Endothelial Cells,” Molecular Vision 19 (2013): 2274–2297. 24265543 PMC3834599

[52] N. V. Strunnikova , A. Maminishkis , J. J. Barb , et al., “Transcriptome Analysis and Molecular Signature of human Retinal Pigment Epithelium,” Human Molecular Genetics 19 (2010): 2468–2486, 10.1093/hmg/ddq129. 20360305 PMC2876890

[53] N. Schäfer , A. Rasras , D. M. Ormenisan , et al., “Complement Factor H‐Related 3 Enhanced Inflammation and Complement Activation in Human RPE Cells,” Frontiers in immunology 12 (2021): 4618, 10.3389/fimmu.2021.769242. PMC8606654 34819935

[54] P. Sánchez‐Corral , R. B. Pouw , M. López‐Trascasa , and M. Józsi , “Self‐Damage Caused by Dysregulation of the Complement Alternative Pathway: Relevance of the Factor H Protein Family,” Frontiers in Immunology 9 (2018): 1607, 10.3389/FIMMU.2018.01607. 30050540 PMC6052053

[55] A. Langford‐Smith , A. J. Day , P. N. Bishop , and S. J. Clark , “Complementing the Sugar Code: Role of GAGs and Sialic Acid in Complement Regulation,” Frontiers in Immunology 6 (2015): 25, 10.3389/fimmu.2015.00025. 25699044 PMC4313701

[56] A. Langford‐Smith , T. D. L. Keenan , S. J. Clark , P. N. Bishop , and A. J. Day , “The Role of Complement in Age‐Related Macular Degeneration: Heparan Sulphate, a ZIP Code for Complement Factor H?,” Journal of Innate Immunity 6 (2014): 407–416, 10.1159/000356513. 24335201 PMC4086042

[57] R. Sharma , A. George , M. Nimmagadda , et al., “Epithelial Phenotype Restoring Drugs Suppress Macular Degeneration Phenotypes in an iPSC Model,” Nature Communications 12 (2021): 1–18, 10.1038/s41467-021-27488-x. PMC8674335 34911940

[58] F. Fenaille , M. Le Mignon , C. Groseil , et al., “Site‐Specific N‐Glycan Characterization of Human Complement Factor H,” Glycobiology 17 (2007): 932–944, 10.1093/glycob/cwm060. 17591618

[59] S. J. Clark , S. McHarg , V. Tilakaratna , N. Brace , and P. N. Bishop , “Bruch's Membrane Compartmentalizes Complement Regulation in the Eye With Implications for Therapeutic Design in Age‐Related Macular Degeneration,” Frontiers in Immunology 8 (2017): 1778, 10.3389/fimmu.2017.01778. 29312308 PMC5742201

[60] B. S. Blaum , J. P. Hannan , A. P. Herbert , D. Kavanagh , D. Uhrín , and T. Stehle , “Structural Basis for Sialic Acid‐Mediated Self‐Recognition by Complement Factor H,” Nature Chemical Biology 11 (2015): 77–82, 10.1038/nchembio.1696. 25402769

[61] K. R. Taylor and R. L. Gallo , “Glycosaminoglycans and Their Proteoglycans: Host‐Associated Molecular Patterns for Initiation and Modulation of Inflammation,” Faseb Journal 20 (2006): 9–22, 10.1096/fj.05-4682rev. 16394262

[62] S. Reitsma , D. W. Slaaf , H. Vink , M. Zandvoort , and M. G. A. Oude Egbrink , “The Endothelial Glycocalyx: Composition, Functions, and Visualization,” Pflugers Archiv: European Journal of Physiology 454 (2007): 345–359, 10.1007/s00424-007-0212-8. 17256154 PMC1915585

[63] S. J. Clark , P. N. Bishop , and A. J. Day , “The Proteoglycan Glycomatrix: A Sugar Microenvironment Essential for Complement Regulation,” Frontiers in Immunology 4 (2013): 1–4, 10.3389/fimmu.2013.00412. 24324472 PMC3840399

[64] J. Hellwage , T. S. Jokiranta , V. Koistinen , O. Vaarala , S. MERI , and P. F. Zipfel , “Functional Properties of Complement Factor H‐Related Proteins FHR‐3 and FHR‐4: Binding to the C3d Region of C3b and Differential Regulation by Heparin,” Febs Letters 462 (1999): 345–352, 10.1016/S0014-5793(99)01554-9. 10622723

[65] J. L. McRae , T. G. Duthy , K. M. Griggs , et al., “Human Factor H‐Related Protein 5 Has Cofactor Activity, Inhibits C3 Convertase Activity, Binds Heparin and C‐Reactive Protein, and Associates With Lipoprotein,” The Journal of Immunology 174 (2005): 6250–6256, 10.4049/jimmunol.174.10.6250. 15879123

[66] J. Tang , F. Woerz , T. Beyer , et al., “Identification of Novel Blood‐Borne Soluble Binding Partners of Factor H‐Related Proteins,” Scientific Reports 16 (2026): 9651, 10.1038/s41598-026-44779-9. 41866380 PMC13009510

[67] M. A. Loeven , M. L. Maciej‐Hulme , C. Yanginlar , et al., “Selective Binding of Heparin/Heparan Sulfate Oligosaccharides to Factor H and Factor H‐Related Proteins: Therapeutic Potential for C3 Glomerulopathies,” Frontiers in immunology 12 (2021): 3322, 10.3389/fimmu.2021.676662. PMC8416517 34489931

[68] S. J. Clark , T. D. L. Keenan , H. L. Fielder , et al., “Mapping the Differential Distribution of Glycosaminoglycans in the Adult Human Retina, Choroid, and Sclera,” Investigative Ophthalmology & Visual Science 52 (2011): 6511, 10.1167/iovs.11-7909. 21746802 PMC3175996

[69] M. Hebecker and M. Józsi , “Factor H‐related Protein 4 Activates Complement by Serving as a Platform for the Assembly of an Alternative Pathway C3 Convertase via Its Interaction With C3b,” Journal of Biological Chemistry (2012): 19528–19536, 10.1074/jbc.M112.364471. 22518841 PMC3365989

[70] H. U. Eberhardt , D. Buhlmann , P. Hortschansky , et al., “Human Factor H‐Related Protein 2 (CFHR2) Regulates Complement Activation Stover CM,” PLoS ONE 8 (2013): e78617, 10.1371/journal.pone.0078617. 24260121 PMC3832495

[71] K. Shi , Z. Wang , Y. Liu , et al., “CFHR1‐Modified Neural Stem Cells Ameliorated Brain Injury in a Mouse Model of Neuromyelitis Optica Spectrum Disorders,” The Journal of Immunology 197 (2016): 3471–3480, 10.4049/jimmunol.1600135. 27671112

[72] Á. I. Csincsi , Z. Szabó , Z. Bánlaki , et al., “FHR‐1 Binds to C‐Reactive Protein and Enhances Rather Than Inhibits Complement Activation,” The Journal of Immunology 199 (2017): 292–303, 10.4049/jimmunol.1600483. 28533443

[73] F. Poppelaars , E. Goicoechea de Jorge , I. Jongerius , et al., “A Family Affair: Addressing the Challenges of Factor H and the Related Proteins,” Frontiers in Immunology 12 (2021): 877, 10.3389/fimmu.2021.660194. PMC8044877 33868311

[74] C. Seguin‐Devaux , J. M. Plesseria , C. Verschueren , et al., “FHR4‐Based Immunoconjugates Direct Complement‐Dependent Cytotoxicity And Phagocytosis Towards HER2‐Positive Cancer Cells,” Molecular Oncology 13 (2019): 2531–2553, 10.1002/1878-0261.12554. 31365168 PMC6887587

[75] Á. I. Csincsi , A. Kopp , M. Zöldi , et al., “Factor H–Related Protein 5 Interacts With Pentraxin 3 and the Extracellular Matrix and Modulates Complement Activation,” The Journal of Immunology 194 (2015): 4963–4973, 10.4049/jimmunol.1403121. 25855355 PMC4416742

[76] M. Mihlan , M. Hebecker , H. M. Dahse , et al., “Human Complement Factor H‐Related Protein 4 Binds and Recruits Native Pentameric C‐Reactive Protein to Necrotic Cells,” Molecular Immunology 46 (2009): 335–344, 10.1016/j.molimm.2008.10.029. 19084272

[77] P. Garred , A. J. Tenner , and T. E. Mollnes , “Therapeutic Targeting of the Complement System: From Rare Diseases to Pandemics,” Pharmacological Reviews 73 (2021): 792–827, 10.1124/pharmrev.120.000072. 33687995 PMC7956994

[78] R. B. Pouw and D. Ricklin , “Tipping the Balance: Intricate Roles of the Complement System in Disease and Therapy,” Seminars in Immunopathology 43 (2021): 757–771, 10.1007/s00281-021-00892-7. 34698894 PMC8547127

[79] M. Cserhalmi , Á. I. Csincsi , Z. Mezei , et al., “The Murine Factor H‐Related Protein FHR‐B Promotes Complement Activation,” Frontiers in Immunology 8 (2017): 1145, 10.3389/fimmu.2017.01145. 28974948 PMC5610720

